# Screening for cognitive impairment among individuals aged 60 years or over: scoping review

**DOI:** 10.1590/1516-3180.2020.0635.150321

**Published:** 2021-07-16

**Authors:** Patrícia Regina Piedade Feichtenberger, Maura Regina Laureano Rocha, Maria Eduarda dos Santos Puga, José Eduardo Martinez

**Affiliations:** I MD. Neurologist and Master’s Degree Student, Pontifícia Universidade Católica de São Paulo (PUC-SP), Sorocaba (SP), Brazil.; II MD, PhD. Speech Therapist, Audiology Specialist and Technical director, FONEC - Fonoaudiologia e Neurociência, Itapetininga (SP), Brazil.; III MD, PhD. Librarian, Information specialist at Cochrane Center in Brazil, São Paulo (SP), Brazil; and Director, Library Network, Universidade Federal de São Paulo (UNIFESP), São Paulo (SP), Brazil.; IV MD, PhD. Rheumatologist and Full Professor, Department of Internal Medicine, Pontifícia Universidade Católica de São Paulo (PUC-SP), Sorocaba (SP), Brazil.

**Keywords:** Cognitive dysfunction, Mass screening, Aged, Dementia, Cognition, Primary care, Elderly, Cognitive impairment

## Abstract

**BACKGROUND:**

Growth in aging of the population has led to increasing numbers of elderly people presenting cognitive impairment and evolution to dementia. There is still no consensus within primary care on the best strategy for screening for cognitive impairment among elderly people. Standardization of a simple but reasonably accurate instrument for a brief cognitive test, in primary care environments, would enable healthcare professionals to identify individuals who require a more in-depth assessment of cognition.

**OBJECTIVES:**

To investigate the instruments used by healthcare professionals in studies conducted worldwide and ascertain the most suitable instruments for screening for cognitive impairment among individuals aged 60 years or over, in the Brazilian population.

**DESIGN AND SETTING:**

Scoping review developed at Pontifícia Universidade Católica de São Paulo, Brazil.

**METHOD:**

A systematic search of the literature was conducted for primary studies using instruments to screen for cognitive impairment among individuals aged 60 years or over, in the MEDLINE, EMBASE, Cochrane Central and LILACS databases.

**RESULTS:**

A total of 983 articles were identified by two independent reviewers, from which 49 were selected for full-text reading, based on the criteria defined for this review. From this, 16 articles adhering to the theme of screening for cognitive impairment among the elderly were selected for in-depth analysis.

**CONCLUSION:**

The Mini-Mental State Examination was the instrument most cited in these studies. The Pfeffer Functional Activities Questionnaire and the Verbal Fluency Test (semantic category) present characteristics favoring further studies, for testing as screening instruments for cognitive impairment among elderly people in Brazil.

## INTRODUCTION

Although substantial increases in the numbers of elderly people are now foreseen in all countries, greater growth is expected in developing regions such as Brazil, where the proportions are expected to become 18.8% in 2020 and 29.3% in 2050.^1,2^

Primary care is considered to be the front line for healthcare for the elderly and can provide regular contacts focused on preventing disabilities resulting from chronic health conditions, such as classification of cognitive impairment in this age group.^3^

Healthcare professionals are faced with the challenge of evaluating the limit of normality among elderly people’s cognitive alterations. Within the concept of senescence, they need to differentiate the expected changes for this age group from the pathological conditions of aging that constitute senility. If such conditions are seen at the prodromal stage, reversal or mitigation may still be possible.^4,5^

Development of dementia in elderly people is a measurable risk. Thus, the pathological transition to this, from a mild stage of cognitive impairment, forms a “gray zone” between normality and initial dementia.^4^

Screening for cognitive impairment among elderly people can be achieved through instruments that have already been translated and validated for application in Brazil.^6^

Bustamante et al.^7^ suggested that cognitive tests and functional scales should be used in combination, in populations with educational heterogeneity. This would improve the accuracy of cognitive screening among mild to moderate cases of dementia because, when used together, they bring more information than when used separately. The functional scales of questionnaires are less influenced by the interviewee’s age, education level or other sociocultural factors.^7^

So far, there is no consensus regarding the best strategy within primary care for screening for cognitive impairment among elderly patients. However, several brief instruments for screening for cognitive impairment have been recommended.^8,9^

No specific drug therapy for treating mild cognitive impairment (MCI) is currently approved. Nonetheless, it was recommended through the FINGER (Finnish Intervention Study to Prevent Cognitive Decline and Disability) study that healthy lifestyle factors such as leisure activities, social interaction, cognitive stimulation, Mediterranean diet and regular physical activity, both for elderly people in general and for those with MCI, should be encouraged as possible protectors against neurodegenerative diseases of aging.^10^

Most individuals and their caregivers would rather know about a diagnosis of dementia as early as possible. This knowledge allows such individuals to make decisions regarding future plans while they still have the ability to do so.^11,12^

In Brazil, around 75% of the population receives its medical care through the public healthcare system (Sistema Único de Saúde, SUS). In this, care is centered on general practitioners, who play an increasingly important role in screening for cognitive impairment among elderly people, which is often neglected within primary care. Moreover, many primary care providers have difficulty in diagnosing dementia accurately. Particularly at the mild stage, dementia is poorly recognized.^13,14^

Thus, instruments for cognitive screening that are quick to apply but relatively accurate are needed, so that healthcare professionals working within primary care can identify individuals who may require a more in-depth evaluation of cognition, at an early stage, and refer them to secondary care.^15^

The present study consisted of a scoping review, in which instruments for screening for cognitive impairment that have been used in studies in the literature, as applicable to individuals aged 60 years or over, were assessed.

## OBJECTIVES

To investigate the cognitive screening instruments used by healthcare professionals in studies conducted worldwide and ascertain which of these are most suitable for use in screening for cognitive impairment among individuals aged 60 years or over, in the Brazilian population.

## METHODS

The PICO technique (Population, Intervention, Comparison, Outcome) was used to define the question and the development of the research, as follows:

P: Population aged 60 years or over.
I: Use of a screening instrument for cognitive impairment in this population.
C: Comparison between screening instruments for cognitive impairment in this population.
O: Verification of the most suitable instruments for screening for cognitive impairment among elderly people, in the Brazilian population.

### Design

This study consisted of a scoping review of the literature. It was conducted in accordance with the Preferred Reporting Items for Systematic Reviews and Meta-Analyses (PRISMA) methodology.^16^

### Search strategy

The searches were conducted in June 2020 in following databases: MEDLINE (Medical Literature Analysis and Retrieval System Online); EMBASE (Excerpta Medica Database); Cochrane Library; and LILACS (Literatura Latino-Americana e do Caribe em Ciências da Saúde).

The descriptors were chosen and identified in accordance with the Medical Subject Heading (MeSH) and Descritores em Ciências da Saúde (DeCS) lists of descriptors, as follows: cognitive dysfunction; mass screening; and elderly.

The same search strategies were used in all databases. The search was refined by specifying randomized clinical trial (RCT) and the elderly age group, or studies that included individuals aged 60 years or over, depending on the filter for searching the information sources for articles, as described in **Table 1**. No limit was placed on the date of publication or the languages of these documents. For cases in which an update was found, the latest version was considered.

**Table 1:** Search strategy

DATABASE	STRATEGY	n	ACCESS
MEDLINE	((“Cognitive Dysfunction”[Mesh] OR (cognitive dysfunctions) OR (dysfunction, cognitive) OR (dysfunctions, cognitive) OR (cognitive impairments) OR (cognitive impairment) OR (impairment, cognitive) OR (impairments, cognitive) OR (mild cognitive impairment) OR (cognitive impairment, mild) OR (cognitive impairments, mild) OR (impairment, mild cognitive) OR (impairments, mild cognitive) OR (mild cognitive impairments) OR (mild neurocognitive disorder) OR (disorder, mild neurocognitive) OR (disorders, mild neurocognitive) OR (mild neurocognitive disorder) OR (neurocognitive disorder, mild) OR (eurocognitive disorders, mild) OR (cognitive decline) OR (cognitive declines) OR (decline, cognitive) OR (declines, cognitive) OR (mental deterioration) OR (deterioration, mental) OR (deteriorations, mental) OR (mental deteriorations))) AND (“Mass Screening”[Mesh] OR (mass screenings) OR (screening, mass) OR (screenings, mass) OR Screening*) **Filters:** Randomized Controlled Trial, Humans, Middle Aged + Aged: 45+ years, Middle Aged: 45-64 years, Aged: 65+ years, 80 and over: 80+ years	134	PubMed
EMBASE	((‘cognitive dysfunction’ OR ‘cognitive dysfunctions’ OR ‘dysfunction, cognitive’ OR ‘dysfunctions, cognitive’ OR ‘cognitive impairments’ OR ‘cognitive impairment’ OR ‘impairment, cognitive’ OR ‘impairments, cognitive’ OR ‘mild cognitive impairment’ OR ‘cognitive impairment, mild’ OR ‘cognitive impairments, mild’ OR ‘impairment, mild cognitive’ OR ‘impairments, mild cognitive’ OR ‘mild cognitive impairments’ OR ‘mild neurocognitive disorder’ OR ‘disorder, mild neurocognitive’ OR ‘disorders, mild neurocognitive’ OR ‘mild neurocognitive disorders’ OR ‘neurocognitive disorder, mild’ OR ‘neurocognitive disorders, mild’ OR ‘cognitive decline’ OR ‘cognitive declines’ OR ‘decline, cognitive’ OR ‘declines, cognitive’ OR ‘mental deterioration’ OR ‘deterioration, mental’ OR ‘deteriorations, mental’ OR ‘mental deteriorations’) AND (‘mass screening’ OR ‘mass screenings’ OR ‘screening, mass’ OR ‘screenings, mass’ OR screening*)) AND (‘controlled clinical trial’/de OR ‘randomized controlled trial’/de OR ‘randomized controlled trial topic’/de) AND ([aged]/lim OR [very elderly]/lim)	245	Elsevier
LILACS	(“Disfunção cognitiva” OR “Comprometimento Cognitivo” OR “Comprometimento Cognitivo Leve” OR “Declínio Cognitivo” OR “Deficiências Cognitivas” OR “Deterioração Mental” OR “Distúrbio Neurocognitivo Leve” OR “Transtorno Neurocognitivo Leve”) AND (“Programas de rastreamento” OR “Exame Coletivo” OR “Identificação Sistemática” OR rastreamento OR screening OR “Triagem de Massa”) AND ( db:(“LILACS”) AND limit:(“aged”))	88	Bireme
Cochrane Library	(((Cognitive Dysfunction*) OR (Dysfunction, Cognitive) OR (Dysfunctions, Cognitive) OR (Cognitive Impairments) OR (Cognitive Impairment) OR (Impairment, Cognitive) OR (Impairments, Cognitive) OR (Mild Cognitive Impairment) OR (Cognitive Impairment, Mild) OR (Cognitive Impairments, Mild) OR (Impairment, Mild Cognitive) OR (Impairments, Mild Cognitive) OR (Mild Cognitive Impairments) OR (Mild Neurocognitive Disorder) OR (Disorder, Mild Neurocognitive) OR (Disorders, Mild Neurocognitive) OR (Mild Neurocognitive Disorders) OR (Neurocognitive Disorder, Mild) OR (Neurocognitive Disorders, Mild) OR (Cognitive Decline) OR (Cognitive Declines) OR (Decline, Cognitive) OR (Declines, Cognitive) OR (Mental Deterioration) OR (Deterioration, Mental) OR (Deteriorations, Mental) OR (Mental Deteriorations)) AND ((Mass Screening*) OR (Screening, Mass) OR (Screenings, Mass) OR (Screening*)) and ((AGED) OR (AGED, 80 AND OVER)))	516	Wiley

### Criteria for inclusion in the scoping review

We only included studies that met the following criteria: randomized clinical trials (RCTs) that had been duly registered or observational studies with random sampling; individuals aged 60 years or over who had been recruited from the general population or from primary healthcare attendees, for random sampling, with absence of any reports of presence of pathological conditions or previous treatments; and application of instruments for screening for cognitive impairment and their implications and results. We considered any outcomes that had been assessed and reported by the original authors.

### Selection of studies

The selection process was performed by two authors (MRLR, PRPF), who independently screened all titles and abstracts that had been found through the electronic search. These authors checked their eligibility in relation to the inclusion criteria. Any disagreements in the selection process were resolved through reaching a consensus or by consulting a third author (JEM). To assess the methodological quality of the studies included, the Downs & Black checklist was used,^17^ with adaptation for RCTs and observational studies. For the RCTs, all questions from this tool were used, with a maximum score of 28 points. For the observational studies, the 17 questions from the original list were used, totaling a maximum of 18 points.

## RESULTS

### Selection of articles

**Figure 1:**
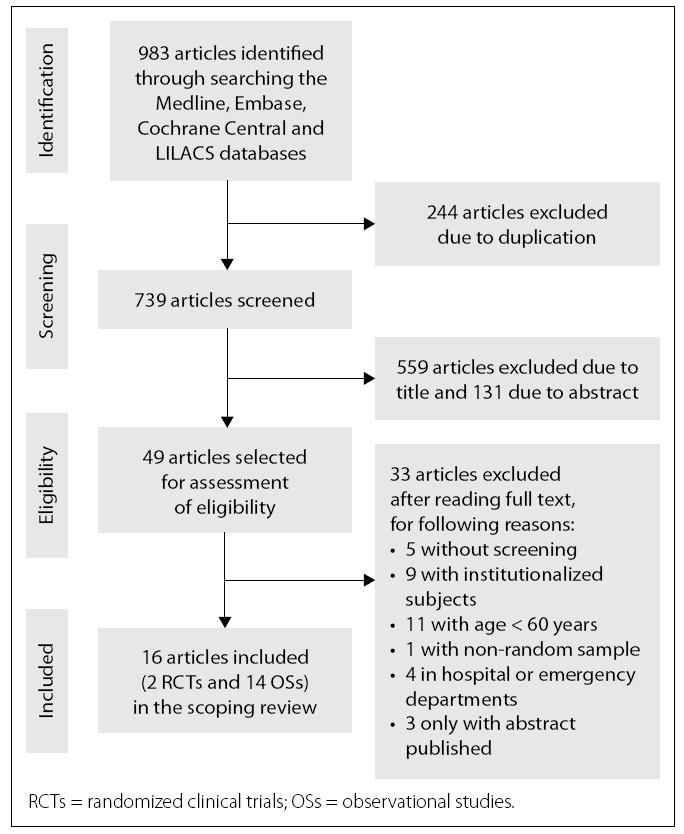
Flowchart for identifying and selecting articles for this scoping review.

We found 983 articles in the first stage of article selection, but 244 articles were excluded due to duplication in the research databases. Thus, 739 articles were retained for assessment of eligibility. In the next phase, articles that did not have the research topic in the title or abstract were excluded. Thus, a further 690 articles were excluded and 49 were selected for assessment of eligibility. Of these, only 16 articles met the objectives of this scoping review (**Figure 1**).

**Tables 2** and **3** provide details on the studies included, so that readers can make their own judgments about the research in these studies.

**Table 2:** Randomized Clinical Trials

Authors, year and country	Study design	Population and setting	Intervention	Comparison	Main findings	Down & Black (maximum score: 28)
Reiner et al.,^20^ 2018; Germany	Cluster-randomized controlled intervention trial; ClinicalTrials. gov identifer NCT01401582	6,440 primary care patients systematically screened for dementia	DemTect and clock drawing test (CDT)	1,601 subjects (41.6% men and 58.4% women); mean age 76 ± 5.3 years (range 70 to 95 years); after DemTect screening, they were assessed using CDT	DemTect is a dementia screening instrument used in Germany with sensitivity of 100% and specificity of 92%; 17.3% (n = 1,117) of the total sample (n = 6,440) were categorized as presenting probable dementia (DemTect score < 9). The sensitivity and specificity of CDT were 84.4% and 45.6% respectively. CDT cannot be regarded as a suitable instrument for detection of probable dementia in primary care. Multi domain tests like DemTect should be considered more appropriate for identifying probable dementia in primary care	25 points/28
Fowler et al.,^18^ 2019; United States	Single-blinded, two-arm, randomized controlled trial; ClinicalTrials. gov identifer NCT01699503	4,005 primary care patients; mean age of the overall study population was 74.1 (± 6.9); 2,256 (66%) were female	Memory impairment screening (MIS) and Mini-Cog	2,008 patients randomized to screening for Alzheimer disease and related dementias (ADRD) and 1,997 patients randomized to no ADRD screening. Primary measurements were health-related quality of life at 12 months and symptoms of depression and anxiety at 1 month	A total of 134 participants (7.7%) in the screening arm screened positive in either MIS or Mini-Cog. Symptoms of depression and anxiety were not harmed through screening and their scores were similar between screened and non-screened ADRD groups. No differences in healthcare utilization, advance planning of care or ADRD recognition by physicians were detected at 12 months	26 points/28

**Table 3:** Observational studies

Authors, year and country	Study design	Population and setting	Inervention	Comparison	Main findings	Down & Black (maximum score: 18)
Morales et al.,^55^1997; Spain	Cross-sectional	Urban sample of 97 subjects (48.5% men and 51.5% women); mean age ± SD = 75.2 ± 6.1 years (range 66-92); 17.9% illiterate. Rural sample of 160 subjects (31.9% men and 68.1% women); mean age ±SD = 73.5 ±8.2 years (range 61 -96); 28.2% illiterate. (Community-dwelling elderly people)	Spanish translation of the Mini-Mental State Examination (MMSE) and Spanish version of the Informant Questionnaire on Cognitive Decline in the Elderly (S-IQCODE)	MMSE and S-IQCODE	Eleven subjects in the urban sample were found to be mild dementia cases (prevalence rate 11.3%) and 23 subjects in the rural sample were dementia cases (prevalence rate 14.4%). S-IQCODE had higher accuracy than MMSE, especially when applied to mild dementia cases, and had higher specificity than MMSE when applied to population-based samples. In the urban sample, S-IQCODE had accuracy of 89% (cutoff ≥ 85 points) versus 77% for MMSE (cutoff ≤ 21 points); and sensitivity of 82% (versus 73% for MMSE). In the rural sample, S-IQCODE had accuracy of 83% (cutoff ≥ 86) versus 75% for MMSE (cutoff ≤ 21); sensitivity was 83% in both tests; and S-IQCODE had specificity of 83% (versus 74% for MMSE)	18 points/18
Dartigues et al.,^66^1997; France	Prospective cohort study	2,726 subjects (59.8% women and 40.2% men); mean age 74.8 ± 6.9 years (range 65-101); 4.3% had never gone to school; 61.2% had a grade school level and 5.7% a university level of education. (Community-dwelling elderly people)	French version of MMSE, Benton's visual retention test (BVRT) and Isaac's set test (1ST)	MMSE < 24, BVRT < 9 and 1ST < 23. Comparison between expected Alzheimer disease (AD) cases was based on the number of low values among the three tests, thus leading to a score of values 0,1,2 or 3	2043 subjects (75%) had at least one complete follow-up screening at 1 or 3 years. At first year of follow-up, 21 subjects were classified as incident cases of dementia (13 as possible or probable Alzheimer's Disease (AD) and 8 as other dementia). At the third year of follow-up, 63 subjects were classified as incident cases of dementia (46 as possible or probable AD and 17 as other dementia). Among the 3 tests (MMSE, BVRT and 1ST), when cutoff level was 1, the sensitivity for diagnosing AD was 90.8% and specificity was 52.2%; When the level was 2, the sensitivity was 81.2% and specificity was 80.4%. When the level was 3, the sensitivity was 52.2% and specificity was 91.3%	18 points/18
Burkart et al.,^35^ 2000; Germany	Cross-sectional	256 subjects. (Community-dwelling elderly people)	A modified German version of the selective reminding procedure (SRP), Mini-Mental State Examination (MMSE) and verbal fluency test (VFT)	MMSE and SRP	23 (9%) of the 256 probands received the diagnosis of dementia (87% female; mean age 87 ± 7.3 years; mean formal education 9.1 ± 1,7 years). MMSE performed better than all SRP scores in terms of sensitivity and specificity. SRP cannot be recommended for dementia screening. MMSE with cutoff of ≤ 23 had sensitivity of 87% and specificity 99%; with cutoff of ≤ 24, sensitivity was 91% and specificity 97%. SRP score cutoffs with specificity of 95% or above had sensitivities below 50%	17 points/18
Silva et al.,^65^ 2002; Sri Lanka	Cross-sectional	380 subjects of mean age 68.2 (SD = 7,17); 33.1% males and 66.9% females; 54.2% had less than six years of formal education; 11.6% had no formal education; and 5.5% were illiterate. (Community-dwelling elderly people)	Translated Sinhalese versions of the MMSE and Cambridge Cognitive Examination (CAMCOG)	MMSE cutoff score ≤ 17 or > 17 points; and CAMCOG	29 of the 33 subjects who screened positive in MMSE showed evidence of dementia. Among the 24 randomly selected subjects who screened negative in MMSE, 22 showed no evidence of dementia while two scored below cutoff in CAMCOG and showed evidence of dementia. The Sinhalese translation of the MMSE is a useful and sensitive instrument for screening for cognitive impairment in Sri Lanka. MMSE cutoff of 19 points had sensitivity of 100% and specificity 84.6%; with cutoff of 17 points, it had sensitivity of 93.5% and specificity 84.6%	15 points/18
Jeong etal.,^64^ 2004; South Korea	Cross-sectional	235 subjects; mean age 73.5 ± 6.7 years; 50.2% had no formal education; and 66.4% were women. (Community-dwelling elderly people)	Korean Mini-Mental State Examination (K-MMSE); Korean version of modified Mini-Mental State Examination (K-mMMSE); Korean version of Informant Questionnaire on Cognitive Decline in the Elderly (IQCODE), using short form of Samsung Dementia Questionnaire (S-SDQ); Korean Instrumental Activities of Daily Living (K-IADL); and Korean version of Expanded Clinical Dementia Rating (CDR)	K-MMSE and K-mMMSE	Among the 235 participants, 46 (19.6%) were classified as having dementia and 54 (22.9%) as having cognitive impairment with no dementia (CIND). K-mMMSE is more sensitive to all levels of CIND and dementia than K-MMSE. At the cutoff of 59/60, K-mMMSE had sensitivity of 0.91 (0.79-0.98) and specificity of 0.78 (0.72-0.84). At the cutoff of 18/19, K-MMSE had sensitivity of 0.91 (0.79-0.98) and specificity of 0.76 (0.69-0.82) to distinguish between demented and normal individuals	16 points/18
Raitetal.,^44^ 2005; United Kingdom	Cross-sectional survey as part of a cluster randomized trial	15,051 subjects; 61.5% were female and 47% were aged between 75 and 79 years. (Community-dwelling elderly people)	Mini-Mental State Examination (MMSE)	MMSE at cutoffs of 23/24 and 17/18	The prevalence of cognitive impairment was 18.3% at cutoff of 23/24 and 3.3% at 17/18 in MMSE	18 points/18
Laks et al.,^63^ 2005; Brazil	Cross-sectional	870 subjects 65.9% were female; 40.1 % were illiterate; 53.4% had 1 -8 years of schooling; 5.3% had 9-11 years of schooling; and 1.2% subjects had more than 12 years of schooling. Mean age 72.14 ±7.26 years. (Community-dwelling elderly people)	Mini-Mental State Examination (MMSE) and Pfeffer Functional Activity Questionnaire (PFAQ)	MMSE and PFAQ	Cognitive and functional impairment was observed in 19.2% of the total sample. Functional impairment without cognitive decline was found in 5.3% of the subjects. Functional impairment was correlated with cognitive impairment. This may be an easier feature for families to recognize and for healthcare professionals to screen for dementia, with assessment of both cognitive and functional status, in combination	18 points/18
Tatsch et al.,^62^ 2006; Brazil	Cross-sectional	1,563 subjects. (Community-dwelling elderly people)	Mini-Mental State Examination (MMSE); Fuld Object Memory Evaluation (FOME); Informant Questionnaire on Cognitive Decline in the Elderly (IQCODE); Activities of Daily Living-International Scale (ADL-IS); Cambridge Examination for Mental Disorders of the Elderly (CAMDEX); Cambridge Cognitive Examination (CAMCOG; cognitive section of CAMDEX); and Clinical Dementia Rating Scale (CDR)	Combination of screening with MMSE, FOME, IQCODE and ADL-IS was tested prior to diagnostic evaluation with CAMDEX, CAMCOG and CDR	Prevalence of dementia was 6.8%. Alzheimer disease and CIND were diagnosed in 64 and 25 subjects, respectively	18 points/18
Ortega et al.,^67^ 2012; Honduras	Cross-sectional	50 subjects; 52% were female and 44% were aged between 71 and 80 years. (Community-dwelling elderly people)	Mini Mental State Examination (MMSE)	MMSE	According to MMSE, 18% were classified as possible cases of dementia	14 points/18
Jiang et al.,^53^ 2014; China	Cross-sectional	1,773 subjects; mean age of participants 72 years; majority female. (Community-dwelling elderly people)	Montreal Cognitive Assessment (MoCA)	MoCA	About 13% (233) of the elderly subjects were identified as having mild cognitive impairment (MCI). The study results suggested that MCI is associated with not doing housework	17 points/18
César et al.,^61^ 2015; Brazil	Cross-sectional epidemiological study	630 subjects; mean age of 71.3 years (± 7.99); range 60-98 years; median 70 years; mean education level 4.9 years (±4.54) with median of 4 years; 14% of participants were illiterate and 28.9% had 1-3 years of education. (Community-dwelling elderly people)	Mini-Mental State Examination (MMSE); Brief Cognitive Screening Battery (BCSB); semantic and phonemic verbal fluency test (VFT); clock drawing test (CDT); Informant Questionnaire on Cognitive Decline in the Elderly (IQCODE); and Pfeffer Functional Activity Questionnaire (PFAQ)	MMSE; BCSB/VFT/ CDT; IQCODE; and PFAQ	110 individuals were diagnosed with dementia and 135 individuals were diagnosed with cognitive impairment without dementia (CIND). The prevalence of dementia found in this study was 17.5% and the prevalence of CIND was 19.5%	18 points/18
Hanetal.,^68^ 2018; South Korea	Prospective cohort study	6,818 subjects; mean age 70.5 ± 7.10 years; 57.5% were women; mean education level was 7.8 ± 5.38 years; illiteracy rate in reading was 4% (n = 275) and illiteracy rate in writing was 4.7% (n = 323). (Community-dwelling elderly people)	Korean version of Short Informant Questionnaire on Cognitive Decline in the Elderly (SIQCODE); Korean version of Consortium to Establish a Registry for Alzheimer's Disease, neuropsychological assessment package (CERAD-K-N); digit span test (DST); executive clock drawing task (CLOX); Frontal Assessment Battery (FAB); Severe Cognitive Impairment Rating Scale (SCIRS); Disability Assessment for Dementia (DAD); and Clinical Dementia Rating (CDR)	The baseline evaluation was conducted over two years from November 2010 to October 2012. Follow-up evaluations were conducted every two years from November to October, from 2012 to 2018	In the baseline evaluation (November 2010 to October 2012), there were 4572 individuals with normal cognition, 1903 individuals with CIND and 343 individuals with dementia	18 points/18
Vega Alonso et al.,^21^ 2018; Spain	Descriptive observational study	4,624 subjects. (Primary care)	Mini-Cog; Mini-Mental State Examination (MMSE); and Alzheimer Questionnaire (AQ)	Mini-Cog screened positive, plus MMSE/AQ	356 patients (8.2%) had a history of dementia or mild cognitive impairment (MCI). Cognitive impairment was confirmed using MMSE or AQ in 67.2% of the cases in which Mini-Cog screened positive.Total number of known cases plus confirmed cases was 806 (18.5%). Prevalence of cognitive impairment was 21.3% among women and 14.8% among men, and it increased with age, reaching maximum values at ages of 85 years or over	18 points/18
César et al.,^32^ 2019; Brazil	Cross-sectional epidemiological study	630 subjects; mean age 71.3 years (± 7.99); range 60-98 years; median 70 years; mean education level was 4.9 years (±4.54) with median of 4 years; 14% of participants were illiterate and 28.9% had 1-3 years of education. (Community-dwelling elderly people)	Montreal Cognitive Assessment (MoCA); Mini-Mental State Examination (MMSE); Brief Cognitive Screening Battery (BCSB); semantic verbal fluency test (VFT), animal category; clock drawing test (CDT); Informant Questionnaire on Cognitive Decline in the Elderly (IQCODE); Pfeffer Functional Activity Questionnaire (PFAQ); and Clinical Dementia Rating (CDR)	MoCA and MMSE total scores stratified into educational levels within each age group	Among the 630 participants, 385 were classified as cognitively normal (CN), 135 as having cognitive impairment with no dementia (CIND) and 110 as having dementia.The MoCA test may not be an adequate tool for identifying individuals with CIND, among those with lower education, but this tool may be used to detect dementia, especially among individuals with more than five years of education, if a lower cutoff score is used, such as 15 points. For MoCA cutoff score 15, CN versus dementia presented sensitivity 90% and specificity 77%; for MoCA cutoff score 19, CN versus CIND presented sensitivity 84% and specificity 49%.	18 points/18

### Results from blinded randomized clinical trials (RCTs)

The RCTs (**Table 2**) were conducted on a total population of 10,445 people, with a weighted average age of 77.49 years. The educational level was only recorded in the study by Fowler et al.^18^

In these studies, four instruments were used, which were all cognitive assessment tests: memory impairment screening (MIS), Mini-Cog, DemTect and clock drawing test^19^ (CDT).

The RCT by Fowler et al.^18^ did not detect any differences in healthcare, quality of life or harm from symptoms of depression and anxiety among individuals who were screened as positive for dementia, through application of MIS or Mini-Cog.^18^

Reiner et al.^20^ compared positive results from cognitive screening using DemTect with the results obtained through the CDT.^19^ They suggested that the CDT^19^ was not a suitable instrument for detection of probable dementia within primary care.^20^

### Results from observational studies (OS)

The sample size in the 14 observational studies (OS) ranged from 50 to 15,051. It was in the range of up to 100 in one article; 101 to 1,000 in seven articles; 1,001 to 10,000 in five articles and more than 10,000 in one article. The total population of the OSs was 35,010 individuals (Table 3).

The participants’ cognitive status was classified as follows: cognitively normal (CN); cognitive impairment with no dementia (CIND); mild cognitive impairment (MCI); and dementia in its respective clinical stages of evolution.

Among the observational studies, 19 instruments (14 cognitive assessment tests and five functional assessment scales) were used to screen the cognition of individuals aged 60 years or over (Tables 4 and 5).

**Table 4:** Cognitive assessment test

Cognitive test	n = sample	Cognitive domains
MoCA^31^ (Montreal Cognitive Assessment)	n = 630^32^ n = 1773^53^	Attention, orientation, language, immediate and delayed memory, constructive praxis, calculation, executive functions, visual-spatial ability
MMSE^34^ (Mini-Mental State Examination)	n = 630^32 61^ n = 4335^21^ n = 1563^62^ n = 870^63^ n = 15051^44^ n = 235^64^ n = 380^65^ n = 256^39^ n = 2792^66^ n = 257^55^ n = 50^67^	Attention, orientation, language, immediate memory, constructive praxis, calculation
BCSB^50^ (Brief Cognitive Screening Battery)	n =630^32 61^	Attention, language, immediate and delayed memory, constructive praxis, executive functions, visual-spatial ability
Mini-Cog^26^	n = 4335^21^	Attention, language, immediate and delayed memory, constructive praxis, executive functions, visual-spatial ability
CERAD^51^ (Consortium to Establish a Registry for Alzheimer’s Disease)	n = 6818^68^	Attention, orientation, language, immediate and delayed memory, constructive praxis, executive functions, visual-spatial ability, calculation
FOME^41^ (Fuld Object Memory Evaluation)	n = 1563^62^	Episodic memory, language, executive functions, learning
CAMCOG^35^ (Cambridge Cognitive Examination)	n = 1563^62^ n = 380^65^	Attention, orientation, language, immediate and delayed memory, constructive praxis, executive functions, visual-spatial ability, calculation
Digit span	n = 6818^68^	Attention, working memory, executive functions, concentration, learning
CDT^19^ (clock drawing test)	n = 6818^68^	Attention, constructive praxis, executive functions, visual-spatial ability
FAB^69^ (Frontal Assessment Battery)	n = 6818^68^	Executive functions
SRP^40^ (selective reminding procedure)	n = 256^39^	Immediate and delayed memory, learning
BVRT^70^ (Benton’s visual retention test)	n = 2792^66^	Memory, executive functions, constructive praxis, visual-spatial functions
IST^36^ (Isaac’s set test)	n = 2792^66^	Language, memory, executive functions
VFT^36^ (verbal fluency test)	n = 256^39^	Language, memory, executive functions

Source: Observational studies.

**Table 5:** Functional evaluation scale

Functional scale	n = sample	Cognitive and functional domains and activities of daily living
IQCODE^54^ (Informant Questionnaire on Cognitive Decline in the Elderly)	n = 630^32 59^ n = 1563^60^ n = 257^53^	Memory, orientation, judgment and problem solving, community affairs, home and hobbies, personal care
PFAQ^37^ (Pfeffer Functional Activity Questionnaire)	n = 630^32 59^ n = 870^61^	Memory, orientation, judgment and problem solving, community affairs, home and hobbies, personal care
CDR^56^ (Clinical Dementia Rating)	n = 630^32 59^ n = 1563^60^	Memory, orientation, judgment and problem solving, community affairs, home and hobbies, personal care
AQ^71^ (Alzheimer’s Questionnaire)	n = 4335^21^	Memory, orientation, judgment and problem solving, community affairs, home and hobbies, personal care
SCIRS^72^ (Severe Cognitive Impairment Rating Scale)	n = 6818^66^	Memory, orientation, motor function, visuospatial function, language

Source: Observational studies.

## DISCUSSION

The criteria used for analysis in this scoping review, on the instruments that might be best suited for use in the Brazilian population, were the following: quick application, validation for use in primary care locations or in the community; adequate psychometric properties; ease of application by members of the healthcare team; the least possible influence from the subject’s educational and cultural level; and whether elderly people’s interest in the evaluation was aroused. The sensitivity and specificity of screening instruments for cognitive impairment among the elderly were also considered.

Use of indiscriminate screening, i.e. for the entire elderly population, irrespective of any cognitive complaints, has been controversial. This is not only because of the need for adaptations to instruments, for them to be applied (given the lack of standardization),^21^ but also because positive results could lead to harm such as anxiety and depression among individuals without any proven dementia. Nonetheless, in the RCT conducted by Fowler et al.,^18^ no harm due to symptoms of depression and anxiety was found after positive screening for dementia.^18^

DemTect^22^, the instrument used by Reiner et al.^20^ is composed of the following tests that are already used in the Brazilian population: immediate memory of a word list, late evocation of the same list, a numerical coding test, a span digit test and a semantic verbal fluency test.^23^ Those researchers did not consider that screening by means of the CDT^19^ to detect probable dementia was an adequate method.^24^ Although the CDT^19^ is easy to apply, it is vulnerable to different interpretations of the final result, given that different ways to analyze the clock that was drawn have been found. It cannot be used among people with visual or motor difficulties that prevent them from properly handling paper and pen, to make the drawing. There is no consensus on whether the CDT^19^ can distinguish MCI from dementia, even though this test can assess memory, motor and executive function and verbal comprehension, and has been shown to differentiate dementia from normal cognition in review studies.^25^

The Mini-Cog^26^ includes the CDT,^19^ with its characteristics as described above, along with immediate and late evocation of three repetitions of words. In the study by Fowler et al.,^18^ Mini-Cog was applied together with MIS. Those authors concluded that Mini-Cog was suitable for routine screening within primary care. However, this test has not been recognized as a good tool for cognitive screening among elderly people in the Brazilian population with less than five years of formal education.^27^

The memory impairment screening (MIS)^28^ test was recommended for use in the Medicare Annual Wellness Visit as a preliminary test in conjunction with other screening tools. It can be effectively applied within four minutes to identify cognitive impairment,^29^ and does not require the ability to write. However, an ability to read is required, which thus means that the results from this test are influenced by the subject’s educational level.^30^

The Montreal Cognitive Assessment (MoCA)^31^ is a test that was designed to screen for MCI and to differentiate it from dementia.^31^ Although it covers all cognitive domains, it is significantly influenced by age and level of formal education. MoCA^31^ may be not an suitable instrument for identifying CIND among individuals with lower levels of education, according to a study by César et al.^32^ However, Cecato et al.^33^ found that MoCA was the test with the highest predictive value for differentiating Alzheimer’s dementia (AD) from MCI and also for differentiating cases of MCI from normal individuals. Furthermore, MoCA has been shown to have significant correlations with the age variable in the mini-mental state examination (MMSE),^34^ Cambridge Cognitive Examination (CAMCOG),^35^ CDT,^19^ verbal fluency test (VFT)^36^ and Pfeffer Functional Activities Questionnaire (PFAQ),^37^ which are instruments that have already been validated and are widely used in the Brazilian population.^33^ Although MoCA^31^ has the disadvantage of taking longer to apply than MMSE^34^ and presents limitations with regard to the capacity for illiterate individuals to perform the proposed tasks, it is a tool that provides a superior overall assessment in the early stages of cognitive decline.^38^

Burkart et al.^39^ compared the selective reminding procedure^40^ (SRP) with MMSE^34^ and concluded that the SRP was not recommendable for cognitive screening for dementia.^40^ The Fuld Object Memory Evaluation (FOME)^41^ assesses memory and learning through the SRP and can be applied to elderly people with a low level of formal education. It uses late evocation after distraction and is applied through a semantic VFT.^36^ In Brazil, the only studies found involved a professional trained in psychology as the evaluator of this test.^42,43^

The MMSE^34^ was the instrument most cited and used in this scoping review, thus corroborating other findings reported in the literature.^21,44^ It has been validated for application both in the community and in primary care in many countries, with the aim of increasing the recognition of cognitive impairment. It has been accepted both by patients and by interviewers, even without assessment of executive function.^29^

Despite being widely used in Brazil, MMSE^34^ needs adjustments to its cutoff scores, which are variable, because it can be influenced by age and level of formal education.^45^ It is a screening tool that can be applied rapidly, and it addresses the main cognitive domains with high specificity and sensitivity for dementia. A wide variety of healthcare professionals have the capacity to use it.^46^

The criteria used in the MMSE^34^ make it highly capable of screening for moderate and severe cognitive impairment. However, its ability to signal milder or earlier degrees of cognitive decline is significantly lower. It is not suitable for screening for the initial phases of dementia and can lead to higher rates of false negative results, since it does not evaluate executive function.^47^ Changes in executive function may be present early in cases of dementia syndrome, and even as the only manifestations. It needs to be emphasized that the MMSE^34^ should not be used in isolation to assess cognitive performance.^48,49^

Use of the semantic VFT^36^ was also seen among the studies reviewed. This enables evaluation of language, memory and executive function, through asking individuals to verbally list categories of colors, animals, fruits or cities. Although it was considered separately only in one study, it was used in other articles in this scoping review, within the FOME^41^ and CAMCOG^34^ tests, the Brief Cognitive Screening Battery^50^ (BCSB), the Consortium to Establish a Registry for Alzheimer’s Disease (CERAD)^51^ battery and the MoCA^31^ test (phonology version).

VFT^36^ is a simple test that can easily be applied. It presents screening results that classify cognition with precision comparable to that of MMSE,^34^ given that it is very effective in evaluating executive function and language ability, mainly due to its semantic approach, which seems to require a high level of thought process. The semantic and phonological versions of VFT^36^ can be considered to be indicators of executive functions since this test requires the ability to self-regulate working memory through the ability to search for and retrieve information that is stored in long-term memory. VFT^36^ is considered to be quite accurate for dementia screening and relatively sensitive for assessing earlier stages of cognitive impairment. The levels of resistance or refusal to participate are low because listing words for one minute is not particularly intimidating. This test is free of charge and easy to administer. It does not require any materials other than a device to keep track of the time and a means for recording the number of words produced. VFT^36^ appears to be able to distinguish between individuals with or without normal cognition. Performance in this test may be influenced by the subject’s level of education and age, which therefore needs to be taken into account.^8,52^

Jiang et al.^53^ suggested that changes to instrumental activities of daily living (IADLs) for domestic work may occur in individuals with MCI and, therefore, use of functional scales is also recommendable. Furthermore, according to Rait et al.,^44^/789ilk individuals with CIND presented higher levels of functional deficit than people with intact cognition.

The Informant Questionnaire on Cognitive Decline in the Elderly (IQCODE)^54^ is a questionnaire (via an interview) that is applied to an individual who accompanies a patient. This companion is asked to quantify the patient’s current performance in different activities of daily living (ADLs), in comparison with the same situations 10 years ago. Morales et al.^55^ showed that the IQCODE^54^ scale presented greater precision of results than the MMSE,^34^ in cases of MCI.

PFAQ^37^ was the functional assessment questionnaire that was most used among the studies reviewed here. It was also aimed at accompanying informants, who were asked to answer 10 simple questions about the performance of elderly people regarding their ADLs. These results can provide direct sensitive information, within the primary care setting, regarding the companion’s suspicion that the patient may present dementia. Use of PFAQ^37^ combined with VFT^36^ showed sensitivity of 88.3% and specificity of 76.5% in a study by Jacinto et al.,^5^ thus suggesting that these tests are useful for screening for cognitive impairment among elderly people.

The Clinical Dementia Rating (CDR)^56^ scale assesses behavior and cognition among elderly people and ascertains the degree of dementia when present. It is capable of identifying individuals for whom the criteria for dementia have not yet been established, but who present cognitive impairment. This instrument is divided into six cognitive and behavioral functions, in order to assess the influence that cognitive impairment can have on the functional capacity to perform ADLs.^57^

The articles selected for the present review showed certain limitations. These included the rate of losses and the short follow-up period for the patients in the RCTs.^18,20^ There was also selection bias in the subsample categories, when tests at different times of assessment were compared. Furthermore, there was no reassessment of participants with a negative result from screening for cognitive impairment.^53^ Evaluation of a sample of patients from primary care and not from the community in general was criticized in some studies,^18, 20, 21^ but this met the inclusion criteria of this scoping review. In addition, given that cognitive impairment can begin many years before dementia syndromes are diagnosed,^58^ further studies on cognitive screening among younger individuals are needed. For example, individuals aged 40 to 60, who may or may not have subjective cognitive complaints, could be assessed. The instruments that were relevant in this review, such as MMSE,^34^ VFT^36^ and PFAQ,^37^ could be used in such studies.

## CONCLUSIONS

The MMSE^34^ was the test most frequently found, and its use and limitations were discussed here. The findings from this scoping review suggest that additional studies on the use of the PFAQ,^37^ in combination with the VFT,^36^ for screening for cognitive impairment among elderly people in the Brazilian population, should be conducted. The positive characteristics of these tools include the reliability of their results; the lower influence of the level of formal education, compared with other instruments; and their ease of application. These additional studies should comprise randomized clinical trials and observational studies to assess the application of PFAQ^37^ and VFT^36^ within primary care, given the diversity of educational and cultural levels in Brazil.

It also necessary to create new cognitive screening instruments for future studies, with the characteristics common to the MMSE,^34^ VFT^36^ and PFAQ,^37^ such as ease of application, in order to obtain standardized results. General practitioners within primary care services can then apply such instruments to elderly people, in order to be able to refer them for wide-ranging and timely evaluation in specialized services, when necessary.

In the context of aging of the population, it is important that professionals should screen for cognitive impairment,^59,60^ as a routine procedure within primary healthcare. Through this, preventive interventions can be provided in order to avoid or minimize the negative effects of dementia on elderly people’s health.
